# Experimental Study Based on Box–Behnken Design and Response Surface Methodology for Optimization Proportioning of Activated Lithium Slag Composite Cement-Based Cementitious Materials

**DOI:** 10.3390/ma17112651

**Published:** 2024-05-30

**Authors:** Weixing Shao, Wenhua Zha, Xueyun Zhou, Tao Xu

**Affiliations:** School of Civil and Architectural Engineering, East China University of Technology, Nanchang 310033, China; zyx19u@163.com (X.Z.); xutt223@yeah.net (T.X.)

**Keywords:** lithium slag, Box–Behnken design, response surface method, cementitious materials

## Abstract

Cement-based cementitious materials occupy a central position in the construction industry, but the problem of high carbon dioxide(CO_2_) emissions from cement production has attracted global attention. To meet this challenge, finding low-carbon alternative materials has become a top priority in the research of new building materials. At the same time, the problem of large amounts of lithium slag piling up needs to be solved, and resource utilization has become its potential way out. In this study, the volcanic ash activity of lithium slag was activated by composite activation means of high-temperature calcination and sodium silicate, and it was used as an alternative mix to cement. The Box–Behnken design and response surface method (*BBD-RSM*) was utilized to optimize the ratio of activated lithium slag composite cement-based cementitious materials, and high-performance new solid waste cementitious materials were prepared. The results show that activated lithium slag composite cementitious materials activated lithium slag exhibit excellent performance when activated lithium slag mass fraction is 7.3%, the sodium silicate dosage is 8.8%, and water–solid ratio is 0.6:1. The composite cementitious material under this ratio shows excellent performance, with fluidity 235.69 mm, gelation time 73.54 s, water evolution rate 1.123%, 3d and 28d compressive strengths, respectively, are 11.54 MPa and 22.9 MPa. Compared with ordinary Portland-cement-based cementing materials, the uniaxial compressive strength, modulus of elasticity, and tensile strength at break of activated lithium slag cementitious material solidified body were increased by 34.33%, 36.43%, and 34.98%, and the compressive deformation and tensile deformation were enhanced by 37.78% and 40%. This study not only provides a theoretical basis and experimental foundation for the preparation of new solid waste cementitious materials, but also provides a new solution for the reinforcement of crushed rock bodies in engineering practice, which is of great significance for promoting the low-carbon development of the construction industry.

## 1. Introduction

Cement-based cementitious materials have a wide range application in construction structure reinforcement, geological disaster management, dam failure prevention, underground rock reinforcement, efficient solid waste disposal and other engineering fields, which all highlight the important supporting role of cement as a basic material [1,2]. However, the problem that cement production brings about high carbon dioxide emissions is gradually coming to a prominent position, and it has become an important factor that restricts the development of the construction industry [2,3]. According to statistics, carbon dioxide emissions during the production of cement-based materials account for 5–8% of the global total [4]. Therefore, it is of great practical significance to find new materials that can replace or partially replace cement and reduce carbon emissions to realize the high-quality development of the civil engineering industry as well as the goal of carbon neutrality. 

The utilization of industrial solid waste materials to manufacture new cementitious materials has a broad prospect [5,6,7]. The reuse and recycling of industrial solid waste materials offer a sustainable approach to reducing CO_2_ emissions. By incorporating these materials into various industrial applications, we can not only mitigate the environmental impact of waste disposal but also contribute to achieving carbon neutrality goals. At present, many scholars have extensively studied industrial solid wastes such as fly ash, coal gangue, and slag as cement substitutes. Wang et al. [6], Liu et al. [7], and Hao et al. [8] significantly improved the properties of cementitious materials by using fly ash as a partial substitute for cement through high-temperature treatment methods. Kim et al. [9] and Fu [10] found that the incorporation of fly ash significantly improved the durability, impermeability, and crack resistance of cementitious materials.

Su et al. [11], Ma et al. [12], Sun et al. [13] successfully applied coal gangue in cementitious materials to improve the strength and durability of the materials while significantly reducing the cost.

Liu et al. [14] concluded that green cement mortar with better compressive strength and resistance to chloride ion permeability can be developed by incorporating activated slag in cement mortar. The studies of Gorospe et al. [15], Xiang et al. [16] also showed that the incorporation of slag could improve the sulphate erosion resistance and durability of cementitious materials. These studies not only verified the potential value of industrial solid wastes in resource utilization, but also provided innovative ideas for other industrial solid wastes.

With the rapid development of the new energy vehicle market and the continuous progress of lithium battery technology, lithium dross production has also increased dramatically. Just China’s lithium dross has totaled more than 15 million tons. Currently, the global lithium dross resource utilization rate is only 6%. Most of lithium slag is disposed via conventional methods such as piling and landfill, resulting in serious waste of resources and environmental pollution. Therefore, how to effectively deal with lithium slag has become an urgent problem in China, and even the world.

Lithium slag, as a by-product of lithium carbonate extraction, is mainly composed of oxides, carbonates, and hydroxides, etc., and contains a large amount of silicon, aluminum, iron, calcium, and other oxides. It has similar chemical composition with cement clinker, and its volcanic ash activity is higher than that of gangue, fly ash and other industrial solid wastes, which is expected to be a powerful supplement to cement-based materials [1,17]. Wu et al. [18] pointed out that the incorporation of lithium slag powder in concrete can significantly improve its densification and impermeability, while showing excellent resistance to freeze–thaw cycles, sulfate attack, and carbonation. It has also been pointed out that the incorporation of lithium slag can significantly affect the gelation time and compressive strength of concrete [19,20]. Therefore, it is important to focus on the important influencing factor of lithium slag dosage (mass fraction) in the related research. These research results provide a theoretical basis for the application of lithium slag in cement-based cementitious materials.

Nevertheless, the volcanic ash activity of lithium slag in its original state is still low, which limits its wide application in cementitious materials [21]. To improve the activity of lithium slag, researchers have explored various methods, such as the use of alkali exciters, high-temperature calcination, and high-temperature complex alkali excitation to activate lithium slag [22,23]. Dong et al. [24], Liu et al. [25] found that alkali exciters could effectively promote the dissolution and hydration reactions of silicates and aluminates in lithium slag, which in turn improved the volcanic ash activity of lithium slag and help to improve the compressive strength of lithium-slag-cement-based cementitious materials and their durability performance. In addition, Shen et al. [26] found that alkaline exciter dosing significantly affected the lithium slag activity. Wu [18] and Wang et al. [27] found that high-temperature complex alkali excitation was more effective in improving the activity of lithium slag. In their study, it was pointed out that high-temperature calcination transforms the silicate and aluminate structures in lithium slag, making them more susceptible to react with calcium hydroxide in cement-based cementitious materials. Meanwhile, alkali excitation further promotes the dissolution and hydration reactions of lithium slag by introducing specific alkaline substances. These methods improve the utilization and performance of lithium slag in cementitious materials by altering the physical and chemical properties of lithium slag, making it easier to react with other components in cementitious materials [28,29].

Despite the positive trend of research related to the use of lithium slag as a cement substitute, the research on the use of high-temperature alkali excitation compounding to activate the volcanic ash activity of lithium slag and then prepare high-performance activated lithium slag cement-based cementitious materials is still at an early stage and lacks systematic proportioning and application studies. This study aims to optimize the ratio of hot-activated lithium-slag-sodium silicate composite cement-based cementitious materials using the Box–Behnken design and response surface method, to provide strong theoretical support and practical guidance for the rational resource utilization of lithium slag and the sustainable development of the construction industry.

Box–Behnken design is an efficient experimental design method which can reduce the number of experiments, lower the experimental cost, shorten the experimental time and significantly improve the experimental efficiency under the premise of guaranteeing the amount of information, and the experimental method has been widely used in traditional fields such as chemistry [28,29], biology [30,31], medicine [32,33] and engineering [23,24]. With the continuous development of new materials in recent years, the method also has a promising application in emerging fields such as recycled concrete [34,35] and high-performance composites [3,15,18]. The development of cementitious materials involves the interaction of multiple factors, and the complexity of these factors makes it difficult to meet the needs of traditional single-factor analysis methods, which can be solved by the box-type Behnken design and response surface methodology.

On this basis, the study activated the volcanic ash activity of lithium slag by high-temperature calcination and sodium silicate complex. The activated lithium slag was used as a partial cement replacement admixture. Box–Behnken design and the response surface method was used to optimize the ratio of activated lithium slag-sodium silicate composite cement-based cementitious materials (hereinafter referred to as composite cementitious materials), to prepare a new type of cement-based cementitious materials. The effect of the composite cementitious material in reinforcing crushed rock is compared with that of ordinary silicate cementitious material, which verifies the scientific rationality of the ratio determined in this study. This study is conducive to promoting the wider application of lithium slag in cement-based cementitious materials and provides strong support for the research and development of new materials and technological innovation.

## 2. Materials and Methods

### 2.1. Material Characterization

The active lithium slag and water glass composite cement-based cementitious material prepared in this study is composed of active lithium slag powder, cement, water glass, water-reducing agent and water mixed in a certain proportion.

#### 2.1.1. Lithium Slag

**Figure 1:**
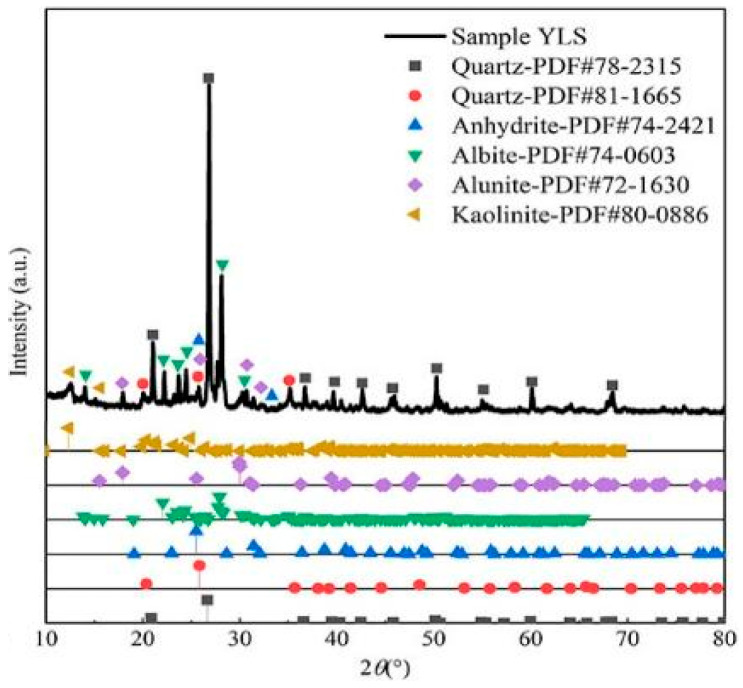
*XRD* pattern of lithium slag.

The lithium slag selected for the test is yellow lithium mica slag, taken from the city of Yichun, Jiangxi Province, China, known as “Asia’s lithium capital”. The specific surface area is 322 m^2^/kg, density is 2.6 kg/m^3^, the median diameter is 19.34 μm, and the amount of water required is less than 115%. The mineral composition measured by *XRD* diffraction analysis is shown in Table 1. The chemical composition of lithium slag is mainly $SiO_{2}$ and $Al_{2}O_{3}$ (active silica-aluminum composition), accounting for 80.92%, indicating that it has high volcanic ash activity. The *XRD* physical analysis spectrum of this lithium slag is shown in Figure 1, and its mineral composition mainly contains gypsum $\left(CaSO_{4}\text{-}2H_{2}O\right)$, quartz, square zeolite, muscovite, and kaolinite, etc. These components can have a secondary hydration reaction with cement hydration product $Ca{\left(OH\right)}_{2}$ in an alkaline environment to generate cement gel, which is conducive to the improvement of the mechanical properties of cement stone. 

In this paper, a simple and easy sieving method was used to sieve lithium slag powder using sieves with different apertures, and the particle content of different particle size ranges was determined by weighing the mass of the residue on different sieves, as shown in Table 2, in which particles smaller than 75 μm accounted for 13.64% of the total, indicating that its specific surface area is larger, which is also the reasons that the volcanic ash activity of lithium residue is higher than that of industrial solid waste such as coal gangue and fly ash.

In this test, lithium slag with a particle size of less than 75 μm was selected as an admixture to replace cement, and these key physical indicators of lithium slag are in line with the industry standard of YBT 4230-2010, “Ground lithium slag used for cement and concrete” [36].

#### 2.1.2. Sodium Silicate 

Sodium silicate, as a commonly used alkali exciter, plays a crucial role in the preparation of gelling materials [13]. The *SP* liquid type sodium silicate was chosen for this experiment.

The modulus and Baumé degrees of sodium silicate are important factors in determining the basic properties of cementitious materials [15,16]. In this experiment, eight types of sodium silicate were selected: modulus was taken as 3.3, and Baume degrees were taken as 30°Bé, 38°Bé, 45°Bé, and 50°Bé; Baume degrees were taken as 38°Bé, and modulus was taken as 2.6, 2.8, 3.0, and 3.3, respectively. 

**Figure 2:**
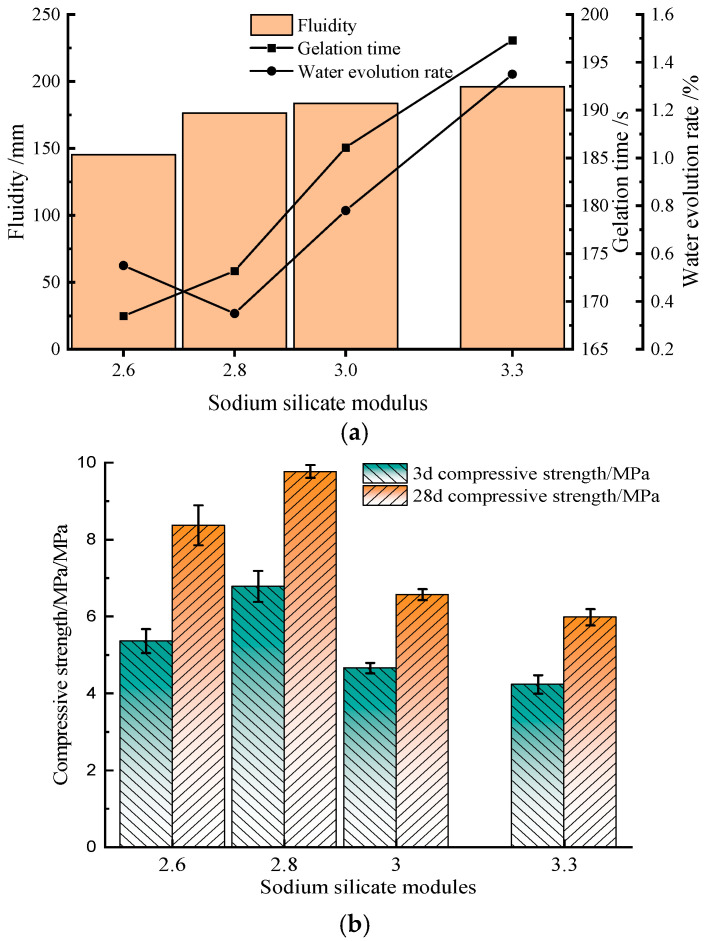
Influence of sodium silicate modulus on the basic properties of composite cementitious. (**a**) Influence of sodium silicate modulus on the fluidity, gelation time and water evolution rate of composite cementitious materials; (**b**) The effect of sodium silicate modulus on 3d and 28d compressive strength of composite cementitious materials.

This study systematically investigated the effects of different types of sodium silicate on the basic properties of composite cementitious materials, such as fluidity, gelation time, water evolution rate and 3d, 28d compressive strength, and the results are shown in Figure 2.

Figure 2a shows as sodium silicate modulus increases from 2.6 to 3.3, fluidity and gelation time of the cementitious material increase, and water evolution rate decreases first and then increases; when the sodium silicate modulus is 2.8, it reaches the minimum value of 0.35% (Meeting engineering requirements). Figure 2b shows 3d and 28d compressive strengths are maximum when sodium silicate modulus is 2.8. Therefore, it is reasonable to choose the water glass modulus of 2.8.

**Figure 3:**
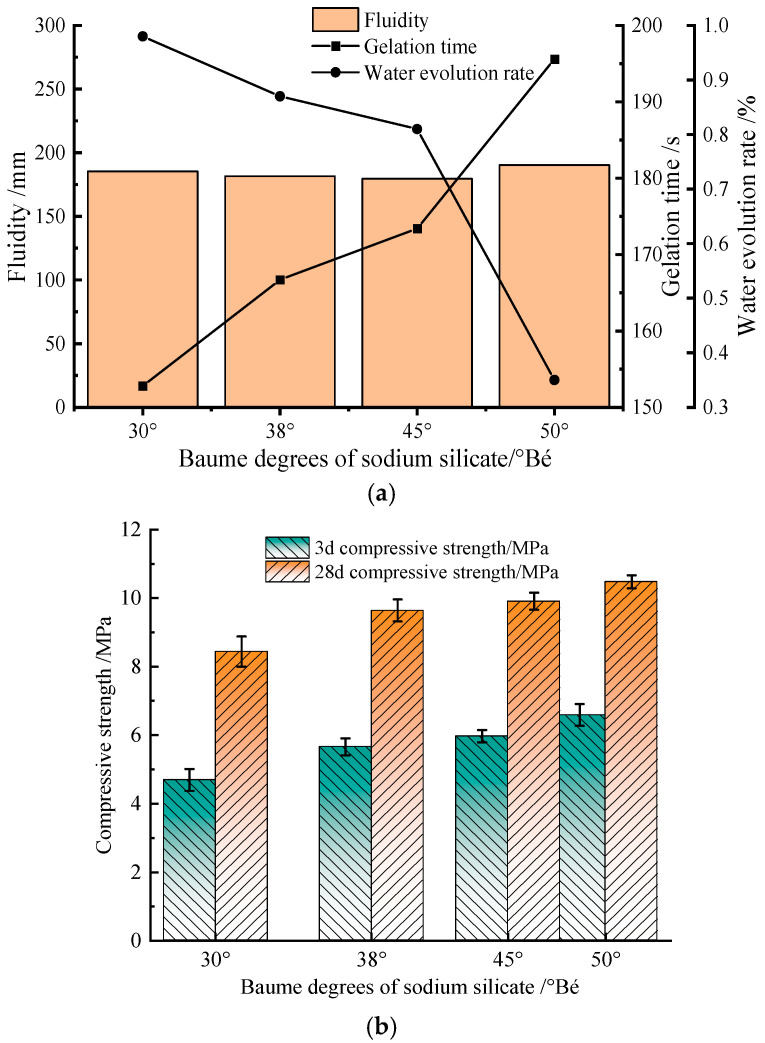
Influence of sodium silicate Baumé degrees on the basic properties of composite cementitious materials. (**a**) Influence of sodium silicate Baumé degrees on fluidity, gelation time and water evolution rate of composite cementitious materials; (**b**) Influence of sodium silicate Baumé degrees on 3d and 28d compressive strength of composite cementitious materials.

Figure 3 shows with sodium silicate Baumé degrees increasing from 30°Bé to 50°Bé, fluidity is almost unaffected, gelation time and compressive strength are gradually increased, and water evolution rate is gradually reduced. When sodium silicate Baumé degrees is 50°Bé, gelation time is about 225 s, and water evolution rate is about 0.35%, which are in line with the requirements of the project [23,25]. Therefore, sodium silicate Baumé degrees is more reasonable at 50°Bé.

#### 2.1.3. Cement

Model P·O 42.5 cement was selected for the test, which is widely used in engineering and has high strength and stability.

#### 2.1.4. Water-Reducing Agent and Water

This test uses polycarboxylic acid water-reducing agent, which is a commonly used cement concrete admixture [19], and the test results of its related indexes are shown in Table 3, and all indicators are in line with the requirements of Test Method for Homogeneity of Concrete Admixtures (GB/T8077-2012) [37] and Concrete Admixtures (GB/T8076-2009) [38]. Water for test is tap water, which has stable water quality and meets the basic requirements for the preparation of cementitious materials.

### 2.2. Experimental Methodology

#### 2.2.1. Lithium Slag Hot Activation Test

To explore the excitation degree of the calcination temperature on lithium slag activity, activation tests at different calcination temperatures were carried out. Firstly, the large lithium slag was crushed and sieved, and the lithium slag powder with a particle size of less than 75 μm was selected, which was evenly spread in the tray and put into the horse-boiling furnace for high-temperature calcination activation.

The experiment was set up with 1 control group and 4 test groups. The control group (*YLS*0) indicates that the doped lithium slag powder was not activated. In test group, lithium slag powder was activated by high-temperature calcination at 600 °C, 700 °C, 800 °C and 900 °C, which were labeled as *YLS*600, *YLS*700, *YLS*800 and *YLS*900, respectively. After reaching the set calcination temperature, the constant temperature was maintained for 2 h, and then the switch of the horse-boiling furnace was turned off, and the slag powder was taken out after it was naturally cooled down. After reaching the set calcination temperature, the constant temperature was kept for 2 h, then the switch of the horse-boiling furnace was turned off, and it was taken out after cooling naturally, and then it was analyzed by *XRD* diffraction to determine the influence of calcination temperature on the activity of lithium slag.

#### 2.2.2. Box–Behnken Design and Response Surface Method (BBD-RSM) Test

In this paper, *BBD-RSM* is used to systematically study activated lithium slag mass fraction, the sodium silicate dosage and the water–solid ratio of three key factors and the interaction between the two factors on the composite cementitious materials, such as fluidity, water evolution rate, gelation time and compressive strength, and finally to obtain the optimal ratio between the influencing factors. The specific steps are as follows:

(1) Set activated lithium slag mass fraction, sodium silicate dosage and water–solid ratio as independent variables, which are, respectively, denoted as $x_{1}$, $x_{2}$ and $x_{3}$. According to engineering practical experience and related research results [35], this paper sets the levels of $x_{1}$ as 5%, 10% and 15%, $x_{2}$ as 6%, 8% and 10%, and $x_{3}$ as 0.6:1, 0.8:1 and 1:1. These indexes directly reflect cementitious material’s engineering performance. Therefore, it is important to optimize them [34,35]. The water–solid ratio is defined as the ratio of the weight of the free water contained in the cementing material to the total weight of cement and lithium slag powder.

(2) To comprehensively reflect the performance characteristics and grouting effect of cementitious materials and optimize the ratio of cementitious materials, fluidity $Y_{1}$, gelation time $Y_{2}$, water evolution rate $Y_{3}$, 3d compressive strength $Y_{4}$, and 28d compressive strength $Y_{5}$ of composite cementitious materials were selected as the response values, and these indexes have a significant influence in the practice of grouting engineering [34,39].

(3) According to the selected factors and levels, *BBD-RSM* was used to design the experimental configurations of three factors, five levels, and three center points, and the coding of independent variables and the level design are shown in Table 4.

Taking the center level in Table 4 as the base group, the level coded values are calculated according to Equation (1):

(1) Xi=(xi−x0)Δx

In the formula: $X_{i}$ is the coded value of the independent variable $x_{i}$. $x_{0}$ is the value of the independent variable xi at the center level. Δx is the step difference between the independent variables.

According to Table 4, 17 groups of experimental schemes were designed as shown in Table 5, and the experimental errors were reduced by setting up multiple repetitive experimental groups (Numbered from 9 to 13).

(4) Prepare composite cement-based grouting materials according to the test program in Table 5, and determine the flow rate by “inverted cone method” and the setting time by “inverted cup method” in accordance with the conventional experimental methods. The water evolution rate was determined by measuring cylinder method, and the uniaxial compressive strength test was carried out for 3d and 28d according to “Test Method for Basic Properties of Ordinary Portland Cement Mortar” JGJ/T70-2009” [40].

#### 2.2.3. Optimal Proportion Verification Test

To verify the reasonableness of optimal proportioning, in this test, grouted consolidated bodies of activation lithium-slag-cement-based cementitious materials (Abbreviations, *ALS*) under optimal proportioning is used as test group, and grouted consolidated bodies of ordinary Portland cement cementitious materials (Abbreviations, *OPC*) is used as control group, and tests of uniaxial compressive strength, modulus of elasticity, and split tensile strength are carried out on their consolidated bodies, respectively. All tests involved are carried out with reference to the Standard for Test Methods of Physical and Mechanical Properties of Concrete (GB/T50081-2019) [41]. The object of grouting reinforcement is selected as coal gangue, a common associated solid waste in coal mining process.

**Figure 4:**
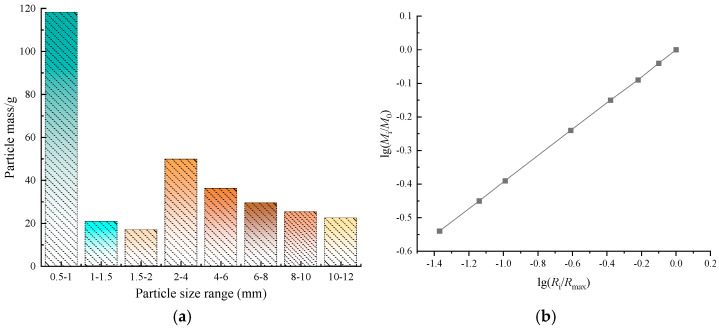
Particle size distribution of coal gangue aggregate. (**a**) Aggregate particle–mass ratio of each particle size range; (**b**) Coal gangue aggregate particle fractal curve.

To reduce the error of indoor test, and to realize the aggregate particle size used in indoor test matched with the particle size of crushed gangue in engineering field, this test configured the crushed gangue pile with the particle size fractal dimension D of 2.5 [35,39]. The crushed aggregate mass in each particle size interval, as shown in Figure 4a. The fractal curves of aggregate particles are shown in Figure 4b. Eq. *M*_i_ is aggregate particles mass in *R*_i_~*R*_min_ particle size interval; *M*_0_ is the total mass of aggregate particles in the *R*_min_~*R*_max_ particle size interval; *R*_i_ is aggregate particles mass with particle sizes not larger than i; *R*_max_ is aggregate mass with the largest particle size.

It can be seen in Figure 4b that the particle–mass ratio within each particle size interval obeys the fractal dimension gradation curve, which meets the uniform description of aggregate particle size distribution [39].

**Figure 5:**
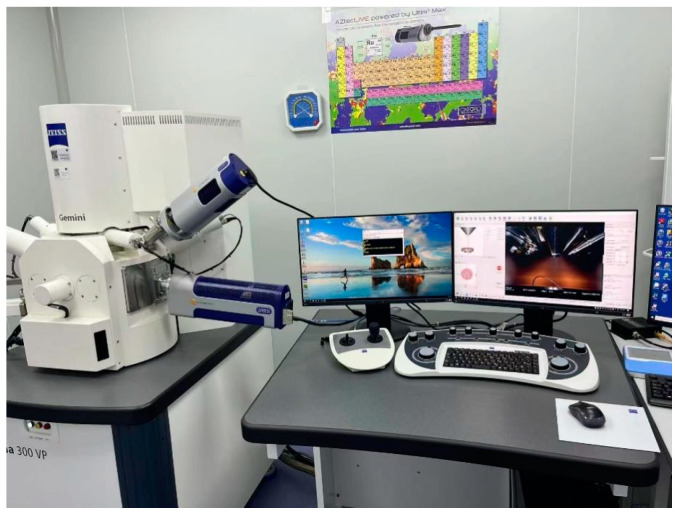
NovaNanoSEM450 field emission scanning electron microscope.

The performance of grouting consolidated body is closely related to its internal structure [23,24]. Therefore, in this study, scanning electron microscope (*SEM*) test was used to observe the microstructural changes and further explore the mechanism of its performance changes. The instrument used in this test is NovaNanoSEM450 field emission scanning electron microscope (FEI company, Hillsboro, OR, USA), as shown in Figure 5.

### 2.3. Summary

**Figure 6:**
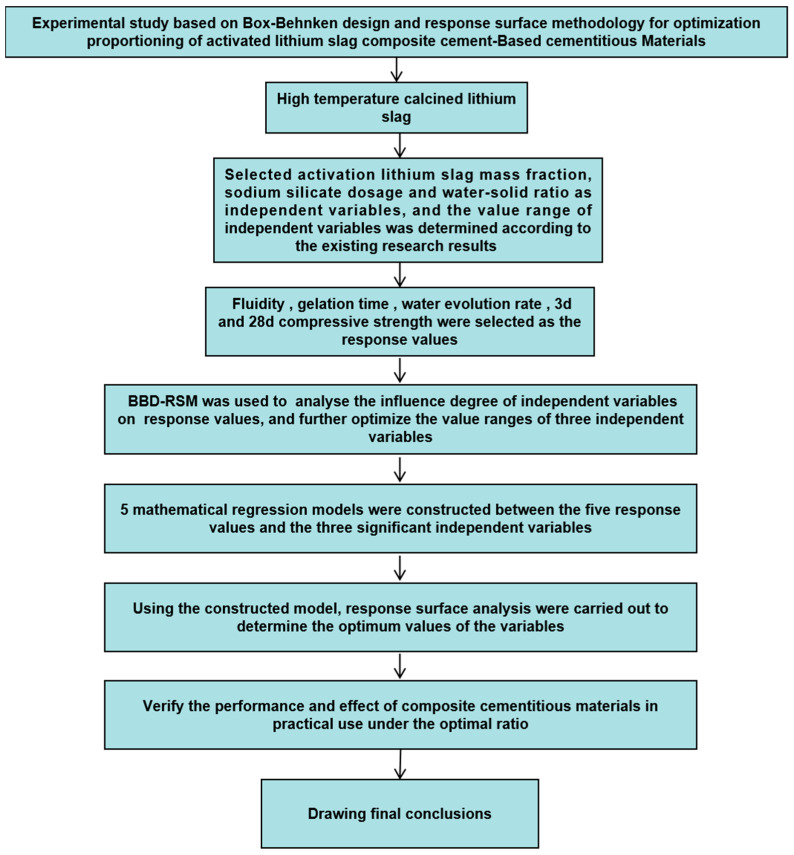
The test flowchart of this chapter.

Figure 6 shows the test flowchart of this chapter. This chapter identifies the raw materials required for the preparation of the composite cementitious materials and describes the tests to be carried out: the lithium slag thermal activation test, which aims to improve the lithium slag volcanic ash activity, the *BBD-RSM* test, which determines the optimal proportioning of the composite cementitious materials, and the validation test under the optimal proportioning. In addition, this chapter presents the correlation between the Box–Behnken Design and response surface methodology (*BBD-RSM)* tests, and the proportion optimization and its mechanism. It is the basic work for the subsequent tests.

## 3. Results and Discussion

### 3.1. Effect of High-Temperature Calcination on the Activity of Lithium Slag

**Figure 7:**
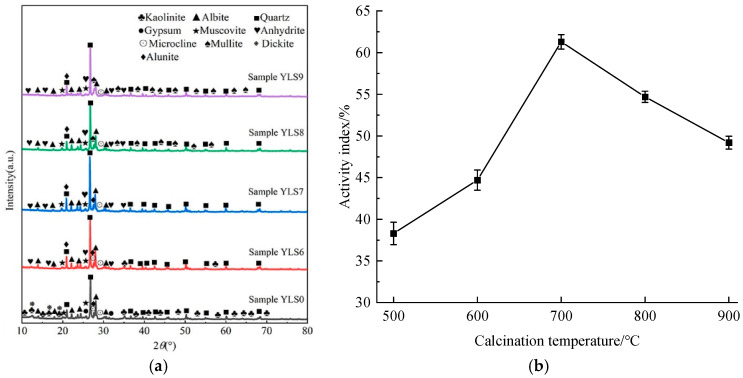
*XRD* patterns and activity index of lithium slag at different calcination temperatures. (**a**) *XRD* patterns; (**b**) activity index.

Figure 7 shows *XRD* patterns of lithium slag at different calcination temperatures. Figure 7a shows that the diffraction peak intensities of kaolinite, sodalite, quartz and alunite in the lithium slag reach the highest at the calcination temperature of 700 °C, and the active components $Al_{2}O_{3}$ and $SiO_{2}$ are the most abundant. After the calcination temperature is higher than 700 °C, amorphous biotite kaolinite generates mullite, which leads to the increase in crystallinity, the dissolution of alunite, and the decrease in active components. Figure 7b also shows that the activity index of lithium slag calcined at 700 °C is the largest. It indicates that the optimal calcination temperature of lithium slag is 700 °C. Therefore, in this study, the lithium slag calcined at 700 °C was selected as an incorporated to replace part of the cement.

### 3.2. Effect of Independent Variables and Their Interaction Terms on Each Response Value

#### 3.2.1. Effects of the Respective Variables on Each Response Value

Test results of 5 response values of lithium slag composite cementitious materials at 17 groups different ratios designed in Table 5 are shown in Table 6.

The 17 groups test results in Table 4 were analyzed and processed using *RDM-BBD*, and the least squares fitted mathematical regression models between 5 response values and the independent variables activated lithium slag mass fraction $x_{1}$, sodium silicate dosage $x_{2}$, and water–solid ratio $x_{3}$ were respectively, constructed using the quadratic model as shown in Equations (2)–(6).

(2) $$Y_{1}=234.90-28.33x_{1}+4.18x_{2}+10.38x_{3}-2.97x_{1}x_{2}-3.63x_{1}x_{3}-1.63x_{2}x_{3}-9.44{x_{1}}^{2}-10.69{x_{2}}^{2}+6.41{x_{3}}^{2}$$

(3) $$Y_{2}=90.77+32.12x_{1}+3.85x_{2}+2.87x_{3}+2.41x_{1}x_{2}+1.59x_{1}x_{3}+1.88x_{2}x_{3}+11.71{x_{1}}^{2}+3.84{x_{2}}^{2}-5.20{x_{3}}^{2}$$

(4) $$Y_{3}=1.10-0.51x_{1}-0.05x_{2}+0.063x_{3}+0.023x_{1}x_{2}-0.21x_{1}x_{3}+0.003x_{2}x_{3}-5.39{x_{1}}^{2}-6.54{x_{2}}^{2}+4.56{x_{3}}^{2}$$

(5) $$Y_{4}=5.92-0.48x_{1}-0.014x_{2}-4.39x_{3}+0.10x_{1}x_{2}-0.37x_{1}x_{3}-0.49x_{2}x_{3}-0.19{x_{1}}^{2}-0.30{x_{2}}^{2}+1.62{x_{3}}^{2}$$

(6) $$Y_{5}=17.35-2.07x_{1}-x_{2}-6.65x_{3}+0.32x_{1}x_{2}-1.72x_{1}x_{3}+0.028x_{2}x_{3}-1.03{x_{1}}^{2}-2.52{x_{2}}^{2}-0.602{x_{3}}^{2}$$

To ensure the reliability of the above five mathematical models, their reliability indexes were verified separately and the results are shown in Table 7.

The coefficient of determination $R^{2}$ indicates the influence degree of independent variables taken on the response values. The larger $R^{2}$, the higher influence is indicated. $R^{2}$ in Table 7 are all greater than 0.95, indicating that the three independent variables have a high influence degree on each response value.

The correction correlation coefficient $R_{adj}^{2}$, indicates the degree to which response value changes can be analyzed by the model. In Table 7, $R_{adj}^{2}$ are all greater than 0.95, indicating that the effect of the independent variables on each response value can be analyzed by this numerical model.

The prediction correlation coefficient, $R_{\mathrm{pre}}^{2}$, indicates the prediction reliability of the model. The $R_{\mathrm{pre}}^{2}$ in Table 7 are all greater than 0.90, and are closer to 1, which shows that the predictive reliability of the 5 constructed response value models is high [34,35].

The signal-to-noise ratio (*SNR*) indicates the reasonableness of the established model. The signal-to-noise ratios in Table 7 are all much greater than 4, indicating that the model constructed for the response values can predict the changes in the response values well [39].

The coefficient of variation (*CV*) indicates how well the regression model fits the sample data points. When it is under 10%, it indicates that the model’s predictions are highly aligned with the observed test data [34,35,39]. The *CV*s in Table 7 are all less than 10%, indicating that the regression model equations fit the test data points well, the error is small, and stability is high [39,42].

To investigate the significance of independent variables and the effect of the interaction terms of independent variables on each response value, this section performs significance tests and *ANOVA* on Equations (2)–(6). *F* values are usually used to compare the significance or not of the differences between groups of factors, and the larger the value, the stronger the significance of the model, which indicates the higher simulation accuracy [43]. *p* values can test the significance of experimental data that are not related to the model [33,44], and in general, the larger *F* value is larger, *p* value is smaller, and when *p* value is <0.05, it means that the effect of the independent variable on the response value is significant. Conversely, it is not significant.

According to the *p* value in Table 7, the significant independent variables of $Y_{1}$ are $x_{1}$ and $x_{3}$, and the significant interaction terms are $x_{1}x_{2}$ and $x_{1}x_{3}$. The significant independent variables of $Y_{2}$ are $x_{1}$, $x_{2}$, $x_{3}$ and the significant interaction terms are $x_{1}x_{2}$, $x_{1}x_{3}$. The significant independent variables of $Y_{3}$ are $x_{1}$ and the significant interaction terms are $x_{1}x_{2}$, $x_{1}x_{3}$. The significant independent variables of $Y_{4}$ are $x_{1}$, $x_{3}$ and the significant interaction terms are $x_{1}x_{3}$, $x_{2}x_{3}$. The significant independent variables of $Y_{5}$ are $x_{1}$, $x_{2}$, $x_{3}$ and the significant interaction terms are $x_{1}x_{2}$, $x_{1}x_{3}$, $x_{2}x_{3}$.

**Figure 8:**
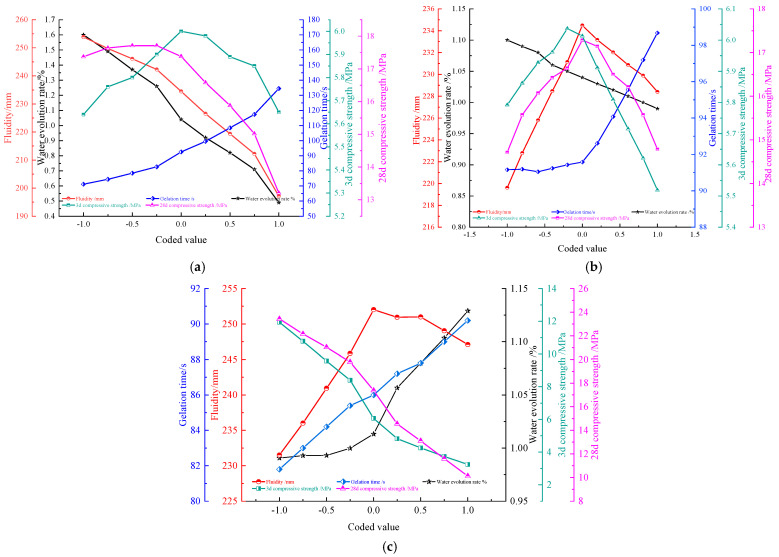
Influence of 3 independent variables on 5 response values. (**a**) The influence of independent variable lithium slag dosage on 5 response values; (**b**) The influence of independent variable sodium silicate dosage on 5 response values; (**c**) The influence of water–solid ratio of independent variable on 5 response values.

Based on the above analyzed results, the effects of three independent variables on five performance indexes of the composite cementitious materials were further predicted according to Equations (2)–(6), and the results are shown in Figure 8.

Figure 8a–c, respectively, shows the effect of activated lithium slag mass fraction $x_{1}$, sodium silicate dosage $x_{2}$, and water–solid ratio $x_{3}$ on fluidity $Y_{1}$, gelation time $Y_{2}$, water evolution rate $Y_{3}$, 3d compressive strength $Y_{4}$ and 28d compressive strength $Y_{5}$.

From Figure 8a, the increase in activated lithium slag mass fraction $x_{1}$ will cause the fluidity $Y_{1}$ and water evolution rate $Y_{3}$ to be greatly reduced, while causing the gelation time $Y_{2}$ to be greatly increased. As activated lithium slag mass fraction $x_{1}$ increases to about 8%, 3d compressive strength, $Y_{4}$, and 28d compressive strength, $Y_{5}$, show a significant increase; as $x_{1}$ continues to increase, $Y_{4}$ and $Y_{5}$ begin to decrease significantly. It was verified that the activated lithium slag mass fraction $x_{1}$ had a significant effect on all five response values, which is consistent with the analytical results in Table 8.

The reason is that the increase in activated lithium slag mass fraction causes the increase in internal surface area of the particles in cementitious materials, and free water is largely adsorbed, resulting in a decrease in fluidity and water evolution rate. At the same time, a smaller mass fraction of activated lithium slag will make lithium slag’s hydration reaction untimely, and internal active ingredient molecules Al-O and Si-O require higher fracture energy, which leads to prolonged chemical reaction time of $SiO_{3}^{2-}$ or $SiO_{4}^{4-}$ and $AlO_{2}^{-}$ with hydration product C-H. The compressive strength of the cementitious material is the greatest at the middle-coded value because the incorporation of activated lithium slag in the early stage accelerated the production of C-H gel. With the continued incorporation of activated lithium slag, the $OH^{-}$ content provided by sodium silicate is relatively reduced, resulting in an inadequate hydration reaction of the lithium slag and a relative reduction in content of the flocculated gel, which ultimately leads to reduced compressive strength. 

From Figure 8b, with $x_{2}$ increasing, $Y_{2}$ of the composite cementitious material shows a significant increasing trend, and $Y_{1}$, $Y_{4}$, and $Y_{5}$ all increase first and then decrease. Among them, $Y_{1}$ and $Y_{4}$ have an overall insignificant magnitude change, while $Y_{5}$ increases or decreases very significantly. $Y_{3}$ decreases slowly with $x_{2}$ increase. This is consistent with the analysis in Table 8.

The reason is, $x_{2}$ increase caused the water content to increase, diluting the grout slurry and leading to $Y_{1}$ of cementitious material to increase. When $x_{2}$ was not in excess, the free water required for the hydration reaction of cement and lithium slag was sufficient, so $Y_{1}$, $Y_{4}$, and $Y_{5}$ also increased. With $x_{2}$ increasing continuously to the middle-coded value, the pores are filled by a large amount of free water, and the excessive $OH^{-}$ ions may interfere the orderly arrangement and polymerization of the molecules of the cementitious constituents, which makes difficult to form the cementation structure, which prolongs the cementation time and leads to a decrease in the compressive strength and water evolution rate.

From Figure 8c, the change rates of $Y_{1}$, $Y_{2}$, $Y_{3}$, $Y_{4}$, and $Y_{5}$ are all approximately linear, indicating that $x_{3}$ increase has a significant effect on each response value. The reason is, $x_{3}$ increase improves the free water content, diluting the slurry, and water evolution rate naturally increases. At the same time, free water fills the voids and the cementation production slows down, making $Y_{2}$ prolonged, $Y_{1}$ larger, and the formation of gel components difficult, leading to a decrease in $Y_{4}$ and $Y_{5}$. Therefore, the water–solid ratio is a key independent variable and must be scientifically and reasonably optimized to obtain the ideal material properties.

#### 3.2.2. Effect of Interaction Term of Independent Variables on Each Response Value

According to Equations (2)–(6), Design-Expert Software (DX13) is used to construct 3D response surfaces, showing the influence of independent variables interaction terms on each response value.

**Figure 9:**
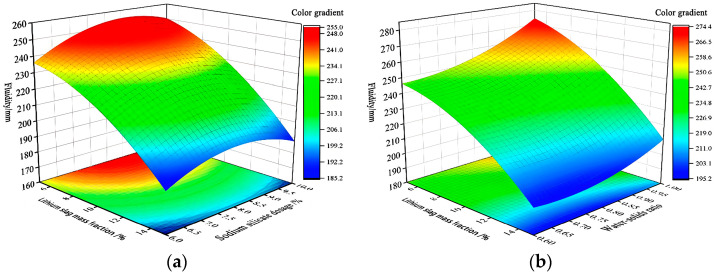
Fluidity 3D response surfaces diagram. (**a**) 3D response surfaces diagram for the influence of the interaction term $x_{1}x_{2}$ on fluidity $Y_{1}$; (**b**) 3D response surfaces diagram for the influence of the interaction term $x_{1}x_{3}$ on fluidity $Y_{1}$.

According to *p*-values in Table 8, it can be seen that the interaction terms $x_{1}x_{2}$ and $x_{1}x_{3}$ are significant influences on the fluidity $Y_{1}$, and according to *p*-values in Table 8, it can be seen that the interaction terms $x_{1}x_{2}$ and $x_{1}x_{3}$ are significant influences on the fluidity $Y_{1}$, and 3D response surfaces are shown in Figure 9, which are parabolic surfaces with openings sloping downward to the ridges. 

Figure 9a shows fixing activation lithium slag mass fraction $x_{1}$, fluidity $Y_{1}$ minimum at sodium silicate dosage $x_{2}$ of 6%. Fixed sodium silicate dosage $x_{2}$, fluidity $Y_{1}$ maximum is at activation lithium slag mass fraction $x_{1}$ of 5%, and minimum is at activation lithium slag mass fraction $x_{1}$ of 10%. According to the engineering requirements of the fluidity $Y_{1}$ [19,20], activation lithium slag mass fraction $x_{1}$ should be taken in (7–8%), i.e., approximately the middle value of its range (5–10%). Figure 9b shows that by fixing water–solid ratio $x_{3}$, fluidity $Y_{1}$ maximum occurs in 8–9% of sodium silicate dosage $x_{2}$.

In summary, activation lithium slag mass fraction $x_{1}$ takes (7–8%), sodium silicate dosage $x_{2}$ is 8–9%, and water–solid ratio $x_{3}$ is 0.6:1; the fluidity $Y_{1}$ is more in line with the engineering requirements.

**Figure 10:**
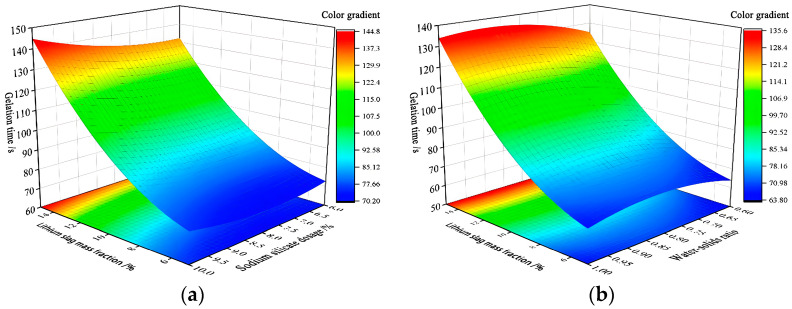
Gelation time response surfaces diagram. (**a**) 3D response surfaces diagram for the influence of the interaction term $x_{1}x_{2}$ on gelation time $Y_{2}$; (**b**) 3D response surfaces diagram for the influence of the interaction term $x_{1}x_{3}$ on gelation time $Y_{2}$.

Three-dimensional response surfaces of gelation time $Y_{2}$ are shown in Figure 10, which are all parabolic surfaces with openings upward or downward to the ridges, proving that $x_{1}x_{2}$ and $x_{1}x_{3}$ have a significant effect on gelation time $Y_{2}$. This is consistent with the analysis conclusion of p-values in Table 8.

Figure 10a shows that fixing sodium silicate dosage $x_{2}$, gelation time $Y_{2}$ is the longest when activated lithium slag mass fraction $x_{1}$ is 15%, and the shortest when it is 5%. When activated lithium slag mass fraction $x_{1}$ exceeds 10%, gelation time $Y_{2}$ will be too long, which will reduce the early strength of the cementitious material, so the optimal value of activated lithium slag mass fraction $x_{1}$ occurs in the range of 5–10%. With fixed activated lithium slag mass fraction $x_{1}$, gelation time $Y_{2}$ is shortest when sodium silicate dosage $x_{2}$ is about 8–9%. Figure 10b shows that fixing sodium silicate dosage $x_{2}$, gelation time $Y_{2}$ is the longest when water–solid ratio $x_{3}$ of 0.6:1 or 0.8:1. Fixing water–solid ratio $x_{3}$, gelation time $Y_{2}$ is the longest when sodium silicate dosage $x_{2}$ of 10%, and the shortest when it is 6%. In summary, when the activated lithium slag mass fraction $x_{1}$ takes (7–8%), sodium silicate dosage $x_{2}$ is 8–9%, and water–solid ratio $x_{3}$ is 0.6:1, gelation time of composite cementitious material will be more reasonable [31,33].

**Figure 11:**
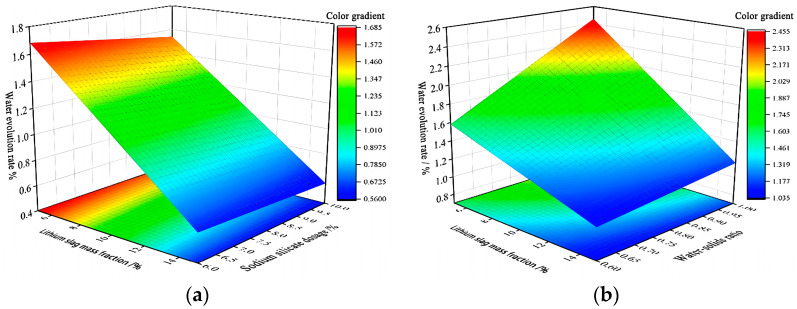
Water evolution rate response surfaces diagram. (**a**) 3D response surfaces diagram for the influence of interaction term $x_{1}x_{2}$ on water evolution rate $Y_{3}$; (**b**) 3D response surfaces diagram for the influence of interaction term $x_{1}x_{3}$ on water evolution rate $Y_{3}$.

Three-dimensional response surfaces of water evolution rate $Y_{3}$ are shown in Figure 11, which are all parabolic surfaces with openings upward or downward to the ridges, proving that $x_{1}x_{2}$ and $x_{1}x_{3}$ have a significant effect on water evolution rate $Y_{3}$. This is consistent with the analysis conclusion of p-values in Table 8.

Figure 11a shows that fixing sodium silicate dosage $x_{2}$ and activated lithium slag mass fraction $x_{1}$ of 6%, the water evolution rate $Y_{3}$ is maximum; fixing activated lithium slag mass fraction $x_{1}$ and sodium silicate dosage $x_{2}$ has almost no effect on water evolution rate $Y_{3}$. Figure 11b shows that fixing sodium silicate dosage $x_{2}$, water evolution rate increases slowly with the increase in water–solid ratio $x_{3}$; sodium silicate dosage $x_{2}$ is 8–9%, and water evolution rate $Y_{3}$ maximum occurs when water–solid ratio $x_{3}$ is 0.6:1.

In summary, the water evolution rate $Y_{3}$ is more in line with the engineering requirements, when activated lithium slag mass fraction $x_{1}$ is in the range of 7–8%, sodium silicate dosage $x_{2}$ is 8–9%, and water–solid ratio $x_{3}$ is 0.6:1 [11,16].

**Figure 12:**
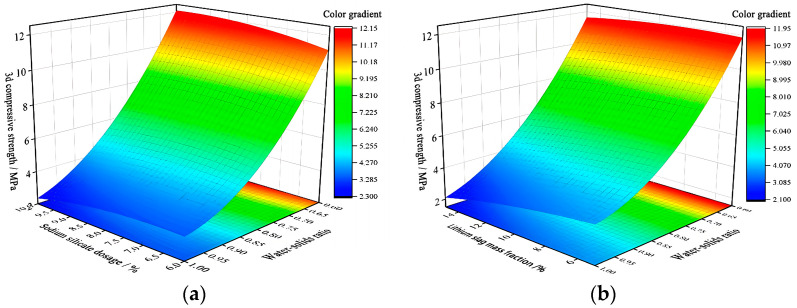
3d compressive strength $Y_{4}$ response surfaces diagram. (**a**) 3D response surfaces diagram for the influence of interaction term $x_{1}x_{3}$ on 3d compressive strength $Y_{4}$; (**b**) 3D response surfaces diagram for the influence of interaction term $x_{2}x_{3}$ on 3d compressive strength $Y_{4}$.

Three-dimensional response surfaces of 3d compressive strength $Y_{4}$ is shown in Figure 12, which are all parabolic surfaces with openings upward to the ridges, proving that $x_{1}x_{3}$ and $x_{2}x_{3}$ have a significant effect on 3d compressive strength $Y_{4}$. This is consistent with the analysis conclusion of p-values in Table 8.

Figure 12a shows that fixing water–solid ratio $x_{3}$, 3d compressive strength firstly increases and then decreases with activated lithium slag mass fraction $x_{1}$ increasing, and reaches the maximum at around 7%. Fixed activated lithium slag mass fraction $x_{1}$, with the increase in water–solid ratio $x_{3}$, 3d compressive strength is decreasing. Figure 12b shows that fixing the water–solid ratio $x_{3}$, the 3d compressive strength $Y_{4}$ increases with the increase in sodium silicate dosage $x_{2}$. Fixed water–solid ratio $x_{3}$, 3d compressive strength reaches its maximum at 8–9% of sodium silicate dosage $x_{2}$.

In summary, when activated lithium slag mass fraction $x_{1}$ is the middle value of (5–10%), sodium silicate dosage $x_{2}$ is at 8–9%, and water–solid ratio $x_{3}$ is 0.6:1, the 3d compressive strength $Y_{4}$ this maximum.

**Figure 13:**
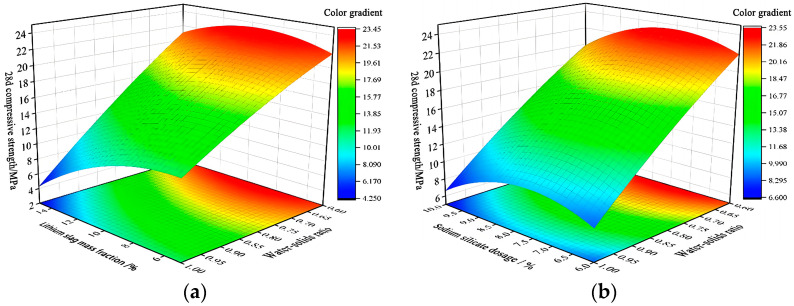
28d compressive strength $Y_{5}$ response surfaces diagram. (**a**) 3D response surfaces diagram for the influence of interaction term on 28d compressive strength $Y_{5}$; (**b**) 3D response surfaces diagram for the influence of interaction term on 28d compressive strength $Y_{5}$.

Three-dimensional response surfaces of 28d compressive strength $Y_{5}$ are shown in Figure 13, which are all parabolic surfaces with openings upward or downward to the ridges, proving that $x_{1}x_{3}$ and $x_{2}x_{3}$ have a significant effect on 28d compressive strength $Y_{5}$. This is consistent with the analysis conclusion of p-values in Table 8.

Figure 13a shows that by fixing water–solid ratio $x_{3}$, 28d compressive strength $Y_{5}$ is maximized at about the middle of its range where activated lithium slag mass fraction $x_{1}$ is (5–10%). Fixing activated lithium slag mass fraction $x_{1}$, 28d compressive strength $Y_{5}$ is maximized at a water–solid ratio $x_{3}$ of 0.6:1. Figure 13b shows that fixing sodium silicate dosage $x_{2}$, 28d compressive strength $Y_{5}$ maximum occurs at a water–solid ratio $x_{3}$ of 0.6:1. Fixing the water–solid ratio $x_{3}$, sodium silicate dosage $x_{2}$ in the range of 8% to 9% maximizes the 28d compressive strength.

In summary, 28d compressive strength $Y_{5}$ is maximized when water–solid ratio $x_{3}$ is taken as the middle value of (5–10%), sodium silicate dosage $x_{2}$ is 8–9% and water–solid ratio $x_{3}$ is 0.6:1.

This section focuses on identifying the key independent variables and their interaction terms that affect the performance indexes of the cementitious materials. Parametric conditions are provided for the analysis of subsequent work

### 3.3. Response Surface Optimal Proportioning Design

According to the effect results of activated lithium slag mass fraction $x_{1}$, sodium silicate dosage $x_{2}$ and water–solid ratio $x_{3}$ on each response value of the composite cementitious material, BBD-RSM was used to regression fit each response value, calculate the optimal parameter values of the numerical regression model, and optimize the multi-parameter and multi-response values. Among them, the optimization range and criteria are shown in Table 9. The optimum ratio was finally obtained to be activated lithium slag mass fraction $x_{1}$ 7.32%, sodium silicate dosage $x_{2}$ of 8.81% and water–solid ratio $x_{3}$ 0.6:1. This result was, respectively, substituted into the mathematical model equations of each response value in Equations (2)–(6), and the results were that fluidity is 235.69 mm, gelation time is 73.54 s, water evolution rate is 1.123%, and 3d and 28d compressive strengths, respectively, are 11.54 MPa and 22.9 MPa.

### 3.4. Optimal Ratio Test Verification Results and Analysis

#### 3.4.1. Mechanical Properties of Grouted Consolidated Body in Crushed Rock Body

**Figure 14:**
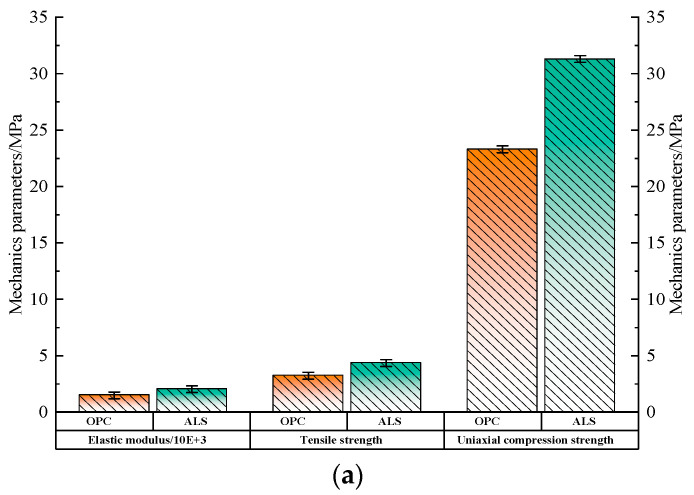

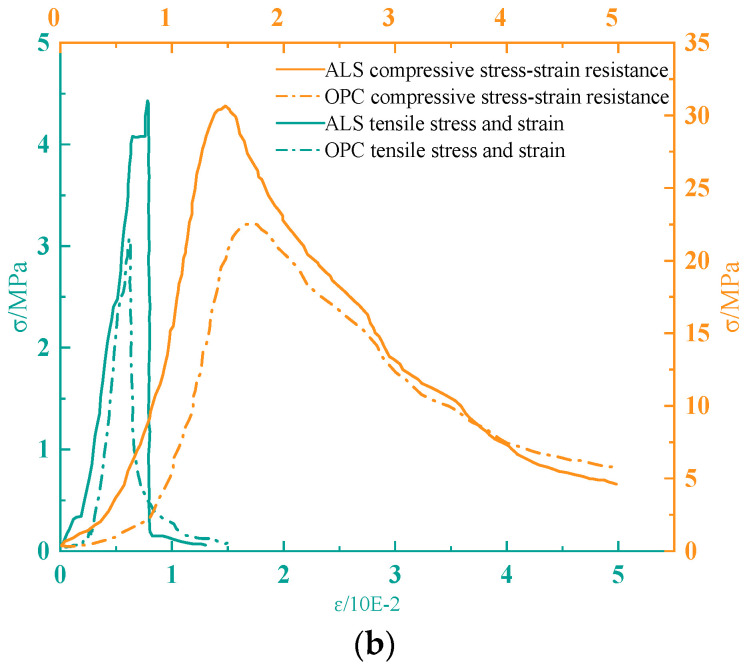
Comparison of mechanical properties of *ALS* and *OPC*. (**a**) Comparison of uniaxial compressive strength, splitting tensile strength and elastic modulus of *ALS* and *OPC*; (**b**) Comparison of stress-strain curves of *ALS* and *OPC*. Abbreviations in figure: ALS—grouted consolidated bodies of activated lithium slag composite cement-based cementitious materials; OPC—grouted consolidated bodies of ordinary Portland cement cementitious materials.

Figure 14a shows mechanical properties of *ALS* and *OPC*. As can be seen from the figure, the compressive strength, elastic model and split tensile strength of *ALS* have been improved by 34.33%, 36.43% and 34.98%, respectively, compared with that of *OPC*.

Figure 14b shows all compressive and tensile stress-strain curves of *ALS* and *OPC* went through a compaction stage, elastic-plastic stage and damage stage. From the figure, it is obvious that compressive and tensile deformation of *ALS* is significantly improved, with the improvement values of 37.78% and 40%, respectively. Figure 14b shows all compressive and tensile stress-strain curves of *ALS* and *OPC* went through a compaction stage, elastic-plastic stage and damage stage. From the figure, it is obvious that compressive and tensile deformation of *ALS* is significantly improved, with the improvement values of 37.78% and 40%, respectively.

#### 3.4.2. Comparison of Macroscopic Rupture Characteristics of Grouted Consolidated Bodies

**Figure 15:**
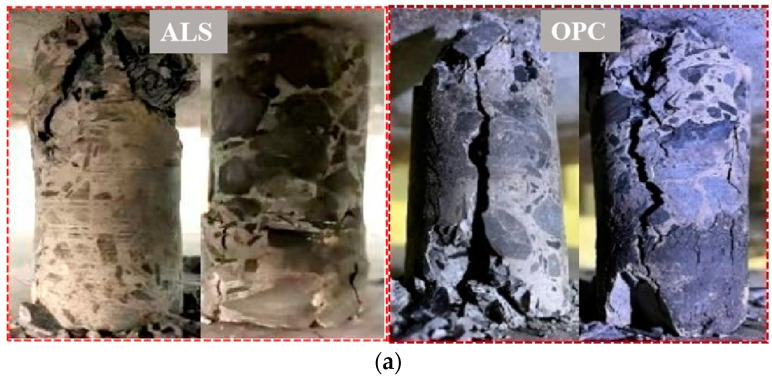

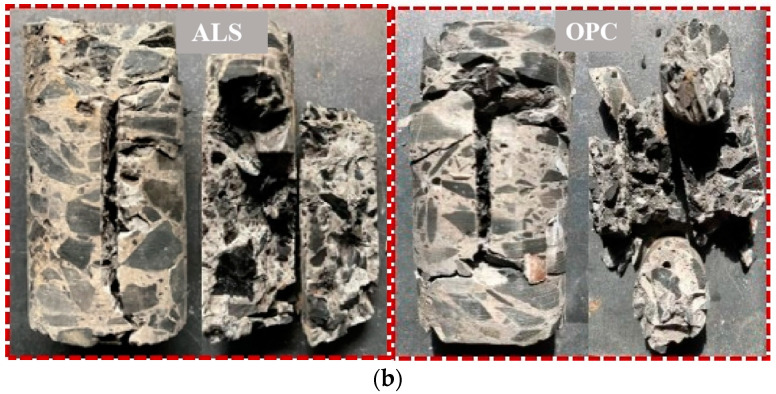
Damage morphology of *ALS* and *OPC*. (**a**) Compression damage pattern of grouted nodular body specimens of *ALS* and *OPC* under uniaxial compression; (**b**) Compressive damage patterns of grouted nodular body specimens from *ALS* and *OPC* under splitting tensile action. Abbreviations in figure: *ALS*—grouted consolidated bodies of activated lithium slag composite cement-based cementitious materials; *OPC*—grouted consolidated bodies of ordinary Portland cement cementitious materials.

Figure 15a shows damage morphology of *ALS* and *OPC* under uniaxial compression. The damage morphology of these two cementitious materials grouted consolidated bodies are basically same in uniaxial compression process, showing “/” type damage morphology. With axial load increasing, micro-cracks were produced in specimen surface, and skin gradually falls off. When axial load reaches the peak pressure, a large number of macro-cracks are rapidly produced internally in the specimen, and these cracks continue to extend and expand until run through, and finally cause damage to the specimen. However, the *ALS* specimen has fewer fine cracks on outer surface under the peak pressure, penetrating cracks are narrower, and the overall damage structure is more complete. The *OPC* specimen has fewer fine cracks, but penetrating cracks are wider and obvious. Therefore, the uniaxial compressive capacity of *ALS* specimens is relatively better.

Figure 15b shows *ALS* specimen splitting tensile damage morphology is relatively single, mainly for the “|” type, the specimen damage degree is low, and the gangue aggregate cemented denser. *OPC* specimen shows multiple penetrating cracks, the damage degree is larger, the internal gangue aggregate pulls off more serious, fracture surface is flatter, and most of the surface is ruptured in the gangue and cementitious materials cementation. Therefore, it is proved that *ALS* has stronger cementing force with gangue aggregate.

#### 3.4.3. Microstructure Characteristics of Grouting Consolidated Body

**Figure 16:**
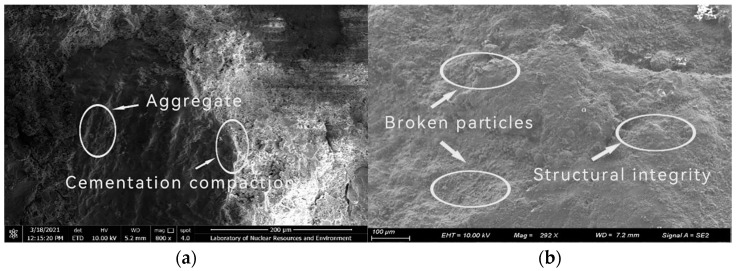
Micro-topography of samples under uniaxial compression. (**a**) ALS micro-topography of samples under uniaxial compression; (**b**) OPC micro-topography of samples under uniaxial compression.

Figure 16 show the *SEM* images of *ALS* and *OPC* samples under uniaxial compression, respectively. It can be seen that the microstructural integrity of *ALS* specimens is higher than that of *OPC* specimens, and their internal structures are denser. It shows that activated lithium slag replaces part of the cement by the alkali excitation of water glass, which can improve the mechanical properties of the solidified body and make the slurry and aggregate denser, and also proves the scientificity of optimal ratios researched out in this paper.

### 3.5. Summary

This chapter determines that the optimal activation temperature of lithium slag is 700 °C. This result is consistent with literature [18,19]. *BBD-RSM* test determines the optimal ratio of lithium slag composite cementitious material as follows: activated lithium slag mass fraction 7.3%, sodium silicate dosage 8.8%, and the water–solid ratio 0.6:1. In addition, this chapter carries out the uniaxial compressive test, modulus of elasticity, splitting tensile strength, and *SEM* test, and verifies that the effect of lithium slag composite cementitious material on grouting reinforcement of crushed gangue is better than ordinary Portland cementitious material, which also verifies that the optimal ratio studied in this paper is scientific, thus supporting the research.

## 4. Conclusions

In this study, based on the *BBD-RSM* test, the ratio of activated lithium slag composite cement-based cementitious material was optimized and the best ratio was obtained. It is also verified that under this optimal ratio, the grouting reinforcement effect of activated lithium-slag-sodium silicate composite cement-based cementitious material on crushed coal gangue is superior to that of ordinary Portland-cement-based cementing materials. The specific contributions and conclusions are as follows:

(1) The optimal calcination temperature of lithium slag was determined to be 700 °C. At this calcination temperature, the content of active components $Al_{2}O_{3}$ and $SiO_{2}$ within lithium slag is the highest. This provides an important reference for improving activity of lithium slag volcanic ash.

(2) The regression mathematical models between active lithium slag mass fraction, water glass dosage and water–solid ratio and the mobility, water evolution rate, gelling time, 3d compressive strength and 28d compressive strength were established, and the accuracy of these regression models was verified. This provides important theoretical basis and reference direction for the ratio optimization of industrial-solid-waste-cement-based composite cementitious materials.

(3) The optimal ratio of lithium-slag-cement-based composite cementitious material was determined as 7.3% of active lithium slag mass fraction, 8.8% of water glass dosage, and water–solid ratio of 0.6:1. With this ratio, the fluidity of this cementitious material was 235.69 mm, gelation time was 73.54 s, water evolution rate was 1.123%, 3d and 28d compressive strengths, respectively, was 11.54 MPa and 22.9 MPa.

(4) The uniaxial compressive strength, modulus of elasticity, and tensile strength at break of activated lithium slag cementitious material solidified body were increased by 34.33%, 36.43%, and 34.98%, and the compressive deformation and tensile deformation were enhanced by 37.78% and 40%, respectively, relative to that of ordinary Portland cement. It is proved that the reinforcing effect of lithium slag composite cementitious material on crushed gangue is better than that of ordinary Portland cement. This finding provides a new type of high-performance cementitious material for underground engineering perimeter rock reinforcement.

## Figures and Tables

**Table 1:** Chemical composition of lithium slag.

Chemical Composition/%	*SiO*_2_	*Al*_2_ *O*_3_	*CaO*	*K*_2_ *O*	*Na*_2_ *O*	*Fe*_2_ *O*_3_	*MgO*	*LOI*
Lithium slag	54.21	26.71	1.30	2.93	0.93	9.26	3.37	17.39

**Table 2:** Basic physical properties of lithium slag.

Original Particle Size/%	<2	<1.25	<1	<0.5	<0.3	<0.25	<0.1	<0.075
Lithium slag	100	63.84	62.21	47.99	32.30	30.99	24.49	13.64

**Table 3:** Performance indexes of naphthalene series water-reducing agent.

20% Liquor PH	Air Dosage/%	Water-Reducing Rate/%	*Cl*^−^/%	Fineness
7~9	≤6	≥45	≤0.3	0.315 mm screen residue <1%

**Table 4:** Box–Behnken design and response surface method (*BBD-RSM*) factor coding and level.

Independent Variable Factor	Code Level		
	−1 (Low Level)	0 (Center Level)	1 (High Level)
$x_{1}$/%	5	10	15
$x_{2}$/%	6	8	10
$x_{3}$	0.6:1	0.8:1	1.0:1

**Table 5:** Box–Behnken design and response surface method (*BBD-RSM*) proportioning design scheme.

**Group**	**1**	**2**	**3**	**4**	**5**	**6**	**7**	**8**	**9**
$x_{1}$/%	−1 (5%)	−1 (5%)	−1 (5%)	−1 (5%)	0 (10%)	0 (10%)	0 (10%)	0 (10%)	0 (10%)
$x_{2}$/%	−1 (6%)	1 (10%)	0 (8%)	0 (10%)	−1 (6%)	1 (10%)	−1 (6%)	1 (10%)	0 (8%)
$x_{3}$	0 (0.8:1)	0 (0.8:1)	−1 (0.6:1)	1 (1:1)	−1 (0.6:1)	−1 (0.6:1)	1 (1:1)	1 (1:1)	0 (0.8:1)
**Group**	**10**	**11**	**12**	**13**	**14**	**15**	**16**	**17**	
$x_{1}$/%	0 (10%)	0 (10%)	0 (10%)	0 (10%)	1 (15%)	1 (15%)	1 (15%)	1 (15%)	
$x_{2}$/%	0 (8%)	0 (8%)	0 (8%)	0 (8%)	−1 (6%)	1 (10%)	0 (8%)	0 (8%)	
$x_{3}$	0 (0.8:1)	0 (0.8:1)	0 (0.8:1)	0 (0.8:1)	0 (0.8:1)	0 (0.8:1)	−1 (0.6:1)	1 (1:1)	

**Table 6:** Design and results of response surface test.

Group	1	2	3	4	5	6	7	8	9	10	11	12	13	14	15	16	17
$Y_{1}$/mm	237.6	256.5	236	276.5	223	230	234.5	235	235	234	235	236	234.5	179	186	194.5	220.5
$Y_{2}$/s	76.8	76.9	61.3	63.7	83.1	89.8	85.3	99.5	90.8	91.2	89.8	90.3	91.7	130.9	140.6	127.6	136.4
$Y_{3}$/%	1.7	1.5	1.4	1.8	1	0.9	1.3	1.2	1.1	1.1	1.1	1.1	1.1	0.6	0.6	0.8	0.3
$Y_{4}$/MPa	6.6	6	11.6	3.3	10.9	12.2	3.3	2.6	5.7	6	6	5.8	6.1	4.7	4.5	12.1	2.4
$Y_{5}$/MPa	16.3	13.7	21.4	12	22.1	20.1	8.3	6.4	17.4	17.1	17.4	17.4	17.5	11.3	9.9	20.9	4.6

**Table 7:** Validation results of the mathematical regression model.

Reliability Index	Fluidity	Gelation Time	Water Evolution Rate	3d Compressive Strength	28d Compressive Strength
$R^{2}$	0.9566	0.9921	0.9674	0.9904	0.9988
$R_{adj}^{2}$	0.9779	0.9819	0.9478	0.9781	0.9974
$R_{\mathrm{pre}}^{2}$	0.9489	0.9766	0.9376	0.9561	0.9843
SNR	13.97	32.45	25.35	27.10	83.42
CV	3.63	3.39	7.01	7.48	1.85

**Table 8:** Analysis of variance results of regression model.

Response Value	Value	$x_{1}/\%$	$x_{2}/\%$	$x_{3}$	$x_{1}x_{2}$	$x_{1}x_{3}$	$x_{2}x_{3}$	$x_{1}^{2}$	$x_{2}^{2}$	$x_{3}^{2}$
$Y_{1}$	F	93.2400	2.0300	12.5100	0.5143	0.7636	0.1534	5.45	6.99	2.52
	P	<0.0001	0.1977	0.0095	0.0465	0.7069	0.0112	0.0523	0.0333	0.1568
$Y_{2}$	F	782.930	11.2200	6.2700	2.2000	0.9535	1.3400	54.74	5.90	10.80
	P	<0.0001	0.0923	0.0508	0.0116	0.0614	0.045	0.0001	0.0455	0.0134
$Y_{3}$	F	267.560	2.5700	4.0200	0.2604	22.150	0.0032	6.7341	5.4341	3.514
	P	<0.0001	0.1399	0.0728	0.0209	0.9559	0.0008	0.0002	0.0437	0.1143
$Y_{4}$	F	8.0000	0.0065	661.70	0.1718	2.2900	4.1700	0.6442	1.59	47.38
	P	0.0255	0.9380	<0.0001	0.0509	0.0471	0.0056	0.4486	0.2481	0.0002
$Y_{5}$	F	452.18	104.61	4664.600	5.4000	156.36	0.0399	228.38	353.40	20.07
	P	<0.0001	<0.0001	<0.0001	0.0532	<0.0001	0.0474	<0.0001	<0.0001	0.0029

**Table 9:** Response optimization standards.

		Optimization Objective	Low Level	High Level	Importance
Independent variable	$x_{1}$/%	Range	5	15	+++
	$x_{2}$/%	Range	6	10	+++
	$x_{3}$	Range	0.6	1	+++
Response value	$Y_{1}$/mm	Maximum	179	276.5	+++
	$Y_{2}$/s	Minimum	61.33	140.66	+++
	$Y_{3}$/%	Minimum	0.33	1.79	+++
	$Y_{4}$/MPa	Maximum	2.4	12.2	+++
	$Y_{5}$/MPa	Maximum	4.59	22.1	+++

Note: ‘+++’ indicate ‘significant’.

## Data Availability

The data presented in this study are available upon request from the corresponding author due to [privacy]. Requests for data access should be directed to the corresponding author at [shaoweixing99@ecut.edu.cn].

## References

[1] Rahman S.M.A., Dodd A., Khair S., Shaikh F.U.A., Sarker P.K., Hosan A. (2023). Assessment of lithium slag as a supplementary cementitious material: Pozzolanic activity and microstructure development. Cem. Concr. Compos..

[2] Hao Y., Guo X., Yao X., Han R., Li L., Zhang M. (2022). Using Chinese coal gangue as an ecological aggregate and its modification: A review. Materials.

[3] Zhang W., Dong C., Huang P., Sun Q., Li M., Chai J. (2020). Experimental study on the characteristics of activated coal gangue and coal gangue-based geopolymer. Energies.

[4] Gu X., Wang H., Zhu Z., Liu J., Xu X., Wang Q. (2023). Synergistic effect, and mechanism of lithium slag on mechanical properties and microstructure of steel slag-cement system. Constr. Build. Mater..

[5] Liu R.Q., Yang Y.Q., Sun S.H. (2018). Effect of M/P and borax on the hydration properties of magnesium potassium phosphate cement blended with large volume of fly ash. J. Wuhan Univ. Technol. (Mater. Ence Ed.).

[6] Wang P., Mao X., Chen S.E. (2019). CO_2_ sequestration characteristics in the cementitious material based on gangue backfilling mining method. J. China Univ. Min. Technol..

[7] Liu Y.J., Wang Z.J., Li R.F., Xu R.L. (2022). Optimization of the pozzolanic activity of coal Gangue Waste for Eco-Efficient Cementitious Materials. Key Eng. Mater..

[8] Hao R., Li X., Xu P., Liu Q. (2022). Thermal activation and structural transformation mechanism of kaolinitic coal gangue from Jungar coalfield, Inner Mongolia, China. Appl. Clay Sci..

[9] Kim T., Davis J.M., Ley M.T., Kang S., Amrollahi P. (2018). Fly ash particle characterization for predicting concrete compressive strength. Constr Build Mater..

[10] Fu C.Q., Ling Y.F., Ye H.L., Jin X. (2021). Chloride resistance and binding capacity of cementitious materials containing high volumes of fly ash and slag. Mag. Concr. Res..

[11] Su Z.N., Li X.H., Zhang Q. (2022). Influence of thermally activated coal gangue powder on the structure of the interfacial transition zone in concrete. J. Clean. Prod..

[12] Ma H.Q., Zhu H.G., Wu C., Chen H., Sun J., Liu J. (2020). Study on compressive strength and durability of alkali-activated coal gangue-slag concrete and its mechanism. Powder Technol..

[13] Sun C., Zhang J., Yan C., Yin L., Wang X., Liu S. (2022). Hydration characteristics of low carbon cementitious materials with multiple solid wastes. Constr. Build. Mater..

[14] Liu J., Yu Q., Zuo Z., Yang F., Han Z., Qin Q. (2019). Reactivity and performance of dry granulation blast furnace slag cement. Cem. Concr. Compos..

[15] Gorospe K., Booya E., Das S. (2022). Effects of supplementary cementitious materials on the durability of glass aggregate mortars. Can. J. Civ. Eng..

[16] Xiang X.D., Xi J.C., Li C.H., Jiang X.-W. (2016). Preparation and application of the cement-free steel slag cementitious material. Constr. Build. Mater..

[17] Wittkowski A., Schirmer T., Qiu H., Goldmann D., Fittschen U.E.A. (2021). Speciation of manganese in a synthetic recycling slag relevant for lithium recycling from lithium-ion batteries. Metals.

[18] Wu F.F. (2016). Study on Hydration Characteristics of Complex Binder Containing Lithium Slag and Properties of hardened concrete. Ph.D. Dissertation.

[19] Dino G.A., Rossetti P., Biglia G., Sapino M.L., Di Mauro F., Särkkä H., Coulon F., Gomes D., Parejo-Bravo L., Zapata Aranda P. (2017). Smart ground project a new approach to data accessibility and Collection for Raw Materials and Secondary Raw Materials in Europe. Environ. Eng. Manag. J..

[20] Yadeta A., Goyal P., Sarkar R. (2023). Modeling the influence of ground-granulated blast furnace slag on hydration of cement. Adv. Civ. Eng..

[21] Shen S.W. (2022). Preparation Mechanism and Performance Research of Multiple Solid Wastes Based Porous Inorganic Polymeric Materials. Ph.D. Dissertation.

[22] Ren C., Wang W., Mao Y., Yuan X., Song Z., Sun J., Zhao X. (2017). Comparative life cycle assessment of sufflaminate clinker production derived from indust rial solid wastes and conventional raw materials. J. Clean. Prod..

[23] Li H., Driesche S.V.D., Bunge F., Yang B., Vellekoop M.J. (2019). Optimization of on-chip bacterial culture conditions using the Box-Behnken design response surface methodology for faster drug susceptibility screening. Talanta.

[24] Dong J.L., Chen L.W., Li L.H., Zhou P., Shi Z., Cai J., Zhang T. (2024). Investigation into the alkali-activation of lithium slag: A sustainable alternative to conventional cement with optimized mechanical properties. Constr. Build. Mater..

[25] Liu Z., Wang J.X., Jiang Q.K., Cheng G., Li L., Kang Y., Wang D. (2019). A green route to sustainable alkali-activated materials by heat and chemical activation of lithium slag. J. Clean. Prod..

[26] Li J., Xu D., Wang X., Wang K., Wang W. (2021). Synergetic–complementary use of industrial solid wastes to prepare high-performance rapid repairmortar. Front. Mater..

[27] Wang Y.R. (2019). Evaluation of Pozzolanic Activity of Lithium Slag and Study on Microstructure Characteristics of Composite Cementitious Materials. Ph.D. Dissertation.

[28] Dai B.B., Zou Y., He Y., Lan M., Kang Q. (2023). Solidification experiment of lithium-slag and fine-tailings based geopolymers. Sustainability.

[29] Li J.Z., Lian P.H., Huang S.W., Huang L. (2020). Recycling of lithium slag extracted from lithium mica by preparing white Portland cement. J. Environ. Manag..

[30] Deng Z. (2022). Utilization of lithium nitrate to mitigate alkali–silica reaction of architectural glass mortar: Characteristics and mechanisms. Constr. Build. Mater..

[31] Wang J., Han L., Liu Z., Wang D. (2020). Setting controlling of lithium slag-based geopolymer by activator and sodium tetraborate as a retarder and its effects on mortar properties. Cem. Concr. Compos..

[32] Zapała-Sławeta J., Owsiak Z. (2016). The role of lithium compounds in mitigating alkali-gravel aggregate reaction. Constr. Build. Mater..

[33] Wang W., Noguchi T. (2020). Alkali-silica reaction (ASR) in the alkali-activated cement (AAC) system: A state-of-the-art review. Constr. Build. Mater..

[34] Li F., Wang Y., Liu K., Wu Y., Ai J., Zhang J. (2022). Preparation of Li_4_SiO_4_-based adsorbents with coal slag for high temperature cyclic CO_2_ capture. Fuel.

[35] Zhang T., Ma B., Tan H., Liu X., Chen P., Luo Z. (2020). Effect of TIPA on mechanical properties and hydration properties of cement-lithium slag system. J. Environ. Manag..

[36] (2010). Ground Lithium Slag Used for Cement and Concrete.

[37] (2012). Methods for Testing Uniformity of Concrete Admixture.

[38] https://law.resource.org/pub/cn/ibr/gb.8076.c.2008.pdf.

[39] Zhou M.Y., Yan J.H., Fan J.Y., Xu Y., Lu Y., Duan P., Zhu Y., Zhang Z., Lu Z. (2023). Insight to workability, compressive strength, and microstructure of lithium slag-steel slag-based cement under standard condition. J. Build. Eng..

[40] (2009). Standard for Test Method of Basic Properties of Construction Mortar.

[41] (2009). Standard for Test Methods of Concrete Physical and Mechanical Properties.

[42] Zulkeplee S.A., Ahmad N.E., Sanusi M.S.M., Hashim S., Ghoshal S.K. (2019). Improved toxic and radioactive elements immobilization in tin slag-blended lithium aluminate borate glass. Radiat. Phys. Chem..

[43] Zang J., Pan C., Li X., Chen K., Chen D. (2023). Research on salt corrosion resistance of lithium-based protective coating on mortar substrate. Materials.

[44] Yang P., Liu L., Suo Y., Xie G., Sun W., Zhang C. (2023). Physical-chemical coupling excitation of low activity coal gasification slag solid waste and its application as a backfill cementitious material. Constr. Build. Mater..

